# Surface plasmon resonance using the catalytic domain of soluble guanylate cyclase allows the detection of enzyme activators^☆^

**DOI:** 10.1016/j.bmcl.2014.01.015

**Published:** 2014-02-15

**Authors:** Filipa Mota, Charles K. Allerston, Kathryn Hampden-Smith, John Garthwaite, David L. Selwood

**Affiliations:** a The Wolfson Institute for Biomedical Research, University College London, Gower Street, London WC1E 6BT, United Kingdom.; b Structural Genomics Consortium, University of Oxford, United Kingdom

**Keywords:** Surface plasmon resonance, Biophysical techniques, Enzyme activators, Soluble guanylate cyclase, Nitric oxide

## Abstract

Soluble Guanylate Cyclase (sGC) is the receptor for the signalling agent nitric oxide (NO) and catalyses the production of the second messenger cyclic guanosine monophosphate (cGMP) from guanosine triphosphate (GTP). The enzyme is an attractive drug target for small molecules that act in the cardiovascular and pulmonary systems, and has also shown to be a potential target in neurological disorders. We have discovered that 5-(indazol-3-yl)-1,2,4-oxadiazoles activate the enzyme in the absence of added NO and shown they bind to the catalytic domain of the enzyme after development of a surface plasmon resonance assay that allows the biophysical detection of intrinsic binding of ligands to the full length sGC and to a construct of the catalytic domain.

## Graphical abstract

**Figure:**
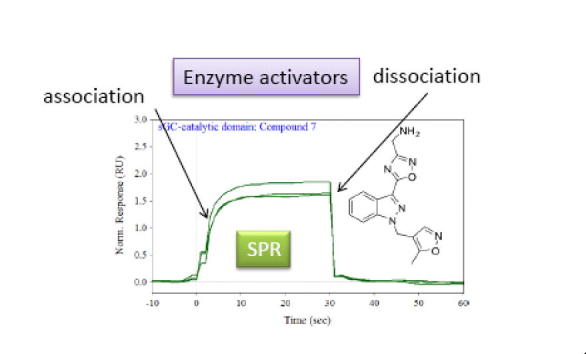

Soluble guanylate cyclase (guanylyl cyclase; sGC; EC 4.6.1.2) is a heterodimeric enzyme endogenously activated by the binding of nitric oxide (NO) to a prosthetic haem group, leading to a conformational change that increases the rate of GTP conversion into cGMP. The second messenger cGMP is a substrate of phosphodiesterases (PDEs), ligand-gated ion channels, and cGMP-dependent protein kinases, resulting in a number of downstream outputs. The NO/sGC/cGMP signalling has an important role in regulating blood flow, and in the perfusion and function of many organs and tissues. Increased levels of cGMP lead to smooth muscle relaxation, inhibition of platelet aggregation and anti-inflammatory effects. Reduced levels or responsiveness to NO contributes to the development of cardiovascular, pulmonary and hepatic diseases. In contrast, aberrant signalling has also been linked to vascular disorders such as hypertension, atherosclerosis and coronary heart diseases and has also been shown to occur during sepsis and neurodegenerative disorders.^1,2^ From a drug design perspective, sGC is an attractive target for small molecule modulators and activators are currently under clinical development for the treatment of cardiovascular diseases.^3,4^ sGC is generally found as a heterodimer composed of a β-subunit (70 kDa) and a larger α-subunit (82 kDa). The best characterized isoform is the α_1_/β_1_ protein, but α_2/_β_1_ is also abundant in the brain.^5^ Each subunit is composed of multiple domains: The haem-nitric oxide binding domain of sGC is located at the N-terminus and Histidine-105 in the β-subunit is the proximal haem ligand; PAS-like domains are present in both subunits and are thought to direct preferential heterodimer formation; turnover of GTP into cGMP occurs at the C-terminus catalytic domain of the enzyme. Whilst the crystal structure of the full length sGC remains unknown, structures of the catalytic domain heterodimer and β-homodimer have been determined (PDB entries 3UVJ and 2WZI, respectively).^6^ The catalytic domain of human sGC possesses two well-defined binding sites, one site responsible for catalysis and one allosteric binding site (also referred to as the pseudosymmetric site), which is thought to be able to accommodate small molecules that control enzyme activity in a manner similar to forskolin in the binding pocket of adenylyl cyclase.^6,7^ It has also been proposed that ATP binds to both sites in sGC and inhibits sGC activity via competition with GTP, highlighting the potential for allosteric regulation of the enzyme.^2,8,9^

There are currently two compound classes known to activate sGC by different mechanisms, one that acts in the absence of NO by replacing the haem moiety, such as BAY 58-2667 (cinaciguat, **1**) and a second class of compounds such as the benzylindazole derivative YC-1 (**2**) which is thought to bind to an allosteric site and act synergistically with NO to increase cGMP production.^10^ Activators derived from YC-1 include the well-characterisedpyrazolopyridine BAY 41-2272 (**3**) (Fig. 1) which acts in a similar fashion.

The mechanisms by which synthetic small molecules activate enzymes differ in many ways from those that apply to inhibitors and it can be challenging not only to discover activators but also to understand their mode of action. Activators commonly bind to an allosteric binding site and require lower doses than inhibitors to cause an effect.^11^ Current biochemical characterisation of sGC modulation by small molecules is performed by measuring cGMP levels with radioimmunoassays. Biophysical characterisation of the enzyme can also be performed using spectroscopy such as Raman and electron paramagnetic resonance.^12,13^ We considered that a biophysical method, surface plasmon resonance, would allow the detection of direct binding of small molecules to sGC and would complement the biochemical characterisation of compounds.

Surface Plasmon Resonance (SPR) is now recognised as a valuable tool to screen and characterise small molecules that modulate the activity of a range of targets, including kinases and G protein-coupled receptors (GPCRs).^14–16^ The microfluidics system of the biosensor allows the injection of the compound over an immobilised protein and the optical detector measures the direct binding between the two entities based on the difference in refractive index near the surface that occurs as the compound binds, thus allowing a real-time and label-free measurement of compound binding to the target. The technique allows the determination of kinetic and thermodynamic parameters. Added benefits of using SPR-based assays include the ability to screen fragments (fragment-based drug design) and the use of separate domains of the protein, which may provide information about the location of the binding site. Further studies with mutated constructs may also allow additional characterisation of the binding mode when crystal structures are not available. We have developed a new assay that measures direct binding of compounds to the sGC enzyme using SPR. The SPR assay was developed to provide a fast means of assaying intrinsic binding of compounds to both the full length human recombinant enzyme and a construct of the catalytic domain only (sGCcat).

The first lead sGC activator was YC-1 (**2**), an indazole derivative that increases cGMP concentration in the presence of NO. YC-1 (**2**) is also capable of inhibiting sodium channels in a voltage-dependent manner, and it was used as the starting point for the design of a library of sodium channel modulators.^17–19^ In this Letter we report cross-screening of this library against sGC and the discovery of new activators of the enzyme.

A subset of 10 drug-like compounds was selected for evaluation (Table 1). Design, synthesis, and full characterisation of these compounds has been reported elsewhere.^19^ The core structure of these compounds is made of an indazole substituted in the 3-position with a 1,2,4-oxadiazole. The main variations in the selected compounds were the substituent groups in the N1 and N2 positions of the indazole (R1) and the groups in the 3-position of the oxadiazole, which included *tert*-butyl carbamate and free-amine groups (R2).

The large heterodimeric structure of sGC makes it particularly challenging to detect binding of low molecular weight compounds using SPR. Whilst higher concentrations of protein on the sensor chip provide a higher signal to noise ratio, a denser surface is also prone to higher non-specific binding and hindrance of the binding site. We evaluated if a construct of the α1β1 catalytic domain of sGC could be used as a model for the detection of binding to sGC. The catalytic domain of sGC (sGCcat) is easier to manipulate in an SPR assay—its smaller size (∼50 kDa) allows the use of a less dense surface (lower immobilisation level), which reduces steric effects and non-specific electrostatic binding whilst still providing a high enough signal/noise ratio.

Both protein constructs, full length and catalytic domain only, were immobilised via amine coupling to a CM5 sensor chip of a Biacore T200 instrument in the presence of ATP and GTP to protect the binding sites.^20^ Expression and purification of the catalytic domain of sGC was performed as previously described except that the protein was finally buffer exchanged in to 50 mM HEPES, pH 7.5, 300 mM NaCl, 5% glycerol, in order to make it ameanable to amine-coupling.^6^ A biochemical assay for sGC activation was also employed. Activation of human recombinant sGC took place in the presence of GTP and the cofactor Mg^2+^, without any added NO.^21^ Enzyme activity was determined by measuring cGMP production using a standard cGMP [^3^H] radioimmunoassay system.^22^

Binding of both nucleotide triphosphates (NTPs) to the immobilised proteins in the SPR assay was observed at physiological concentrations in the presence of Mg^2+^, which was present at 10 mM in the running buffer, whilst binding of cGMP only shifted from the baseline at concentrations higher than 1 mM. Surface saturation could not be observed, as NTPs are known to aggregate at concentrations higher than 1 mM^23^ (Fig. 2). However, binding curves were fit to a nonlinear curve model that considers binding plus linear non-specific binding. The fit was consistent with GTP binding to one binding site (*n* = 1) and ATP binding more than one site (*n* = 1.2) (Fig. 3). The conformation of the catalytic domain of sGC is thought to change significantly upon activation and ‘open’ and ‘closed’ states have been suggested.^6^ Although it is not possible to know in which conformation the protein was immobilised, binding of ATP and GTP shows that binding sites are accessible.

Both proteins tolerated DMSO concentrations of up to 5% which allowed binding of the small molecules to be evaluated using this system. The binding level of all compounds was measured at 100 μM.^24,25^

With realistic responses observed for the nucleotides GTP, cGMP and ATP we next tested known activators in the SPR assay. Binding to the full-length enzyme was non-stoichiometric, indicating there is more than one site where small molecules can bind non-specifically. Nonetheless, YC-1 (**2**) demonstrated strong binding to both proteins indicating its binding site can be located in the catalytic domain, consistent with previous investigations.^26^ In contrast, the heme mimetic BAY 58-2667 (**1**), which is known to bind to the heme domain, showed strong binding to the full length sGC and only non-specific binding to sGCcat, as per analysis of the sensorgrams (see Supplementary information).

Competition between ATP and GTP at 1 mM each was observed, as the biosensor response for binding of both nucleotides together was lower than the sum of their separate binding responses. Conversely, GTP and YC-1 (**2**) showed additive binding, suggesting binding of the small molecule to the pseudosymmetric site of the catalytic domain (Table 1).

We next evaluated the indazoleoxadiazole compound library in the SPR and biochemical assays (Table 2). The binding response of the compounds to sGCcat were converted into percent occupancy, defined as the possible number of immobilised sites occupied by the compound at equilibrium, assuming a stoichiometric interaction between the compound and the enzyme. Compounds with highest affinity would also show higher percent occupancy (Table 1).^27^

Compounds **4** and **5** are close analogues, differing only in the position of a methyl group, and both give a high binding response to sGC in the SPR assay, similar to YC-1 (**2**) itself. The 2-methylbenzyl-substituted indazole **5** does not activate the enzyme in the biochemical assay. Replacement of the methyl group in the 4-position of compound **4** by a methoxy group in compound **6** not only renders the compound inactive but also prevents it from binding to the enzyme in the SPR assay.

Further variations at the N1 substituted indazole include a 5-methylisoxazole in compound **7** and an ethanone in compound **8**. The latter showed low binding to sGC and no activity in the biochemical assay. Compound **7** on the other hand, activated the enzyme and also showed binding in the SPR assay to a lower extent than compound **4**.

The *tert*-butyl carbamate substituted (1,2,4-oxadiazol-3-yl)methanamines **9** and **10** vary in the substituents at the N1 of the indazole core. The 5-ethylbenzo[*d*][1,3]dioxole compound **10** is capable of activating sGC and shows significant binding to the enzyme, whilst the *N*-(4-ethylphenyl)acetamide compound **9**, despite showing higher binding than **10** is inactive in the biochemical assay. The acetamide group, which is both a hydrogen bond donor and acceptor, might contribute to a higher binding affinity, but this is not necessarily required for activity.

Three compounds (**11**–**13**) were selected in which the indazole core was substituted at the N2 position, and whilst compounds **11** and **12** showed binding to sGC they did not exhibit any biological activity. Unfortunately the pyrazolopyridine derivative BAY 41-2272 (**3**) showed poor solubility in the assay buffers even with 5% DMSO and crystallised in the assay plates preventing evaluation.

The binding of the indazoleoxadiazole compounds to the full-length sGC correlates with the binding to the catalytic domain construct, providing evidence that the binding site for this class of compounds is at the catalytic domain of sGC. This is most likely at the pseudosymmetric site, as previously suggested by structural models of YC-1 (**2**) interaction with the catalytic core of sGC and additive binding of the compound with GTP.^26^ No correlation was observed between binding level and lipophilicity (*C* log *P*) of the compounds (shown in Supplementary information). Notably no binding was found for BAY 58-2667 (**1**) to the catalytic domain. The SPR assay detects binding alone and is not expected to correlate with biochemical activation and, based on our data, does not do so. However, binding must be a pre-requisite for activity and we did not observe biochemical activation without binding.

In summary, we have described a new assay that detects intrinsic binding of nucleotides and small molecules to the NO-receptor sGC, and provided further evidence that the so-called NO-independent sGC activators act through binding to an allosteric site on the catalytic domain of the enzyme. We utilised the assay to evaluate a library of YC-1 (**2**) related compounds and discovered some new indazoleoxadiazole activators as a result. The α1β1 sGC catalytic domain construct used in this assay can therefore be used as a model for sGC binding in further SPR-based studies, providing a simple direct method to enable fragment-based drug design and inhibitor screening.

## Figures and Tables

**Figure 1:**
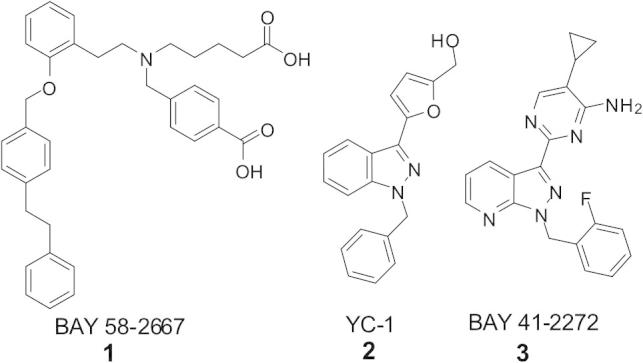
Chemical structure of sGC activators.

**Figure 2:**
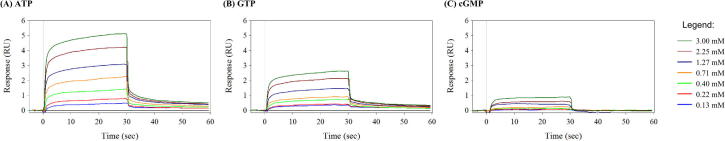
Sensorgrams for ATP (A), GTP (B), and cGMP (C) binding to sGCcat. The binding of the nucleotides at concentrations 0.13–3 mM was monitored for 30 s in the presence of the cofactor Mg^2+^ (*n* = 2).

**Figure 3:**
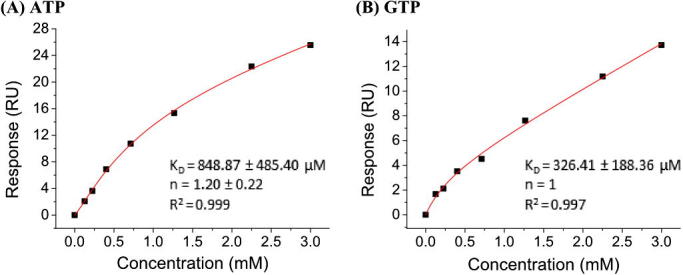
Fitting curves for ATP and GTP binding to sGCcat.(A) ATP binds to the receptor with a stoichiometry of 1.2 and *K*_D_ = 848.87 μM.(B) GTP binds sGCcat with a stoichiometry of **1** and *K*_D_ = 326.41 μM. (*n* = 2).

**Table 1:** Structures of selected compounds tested for sGC activity

**Figure:**
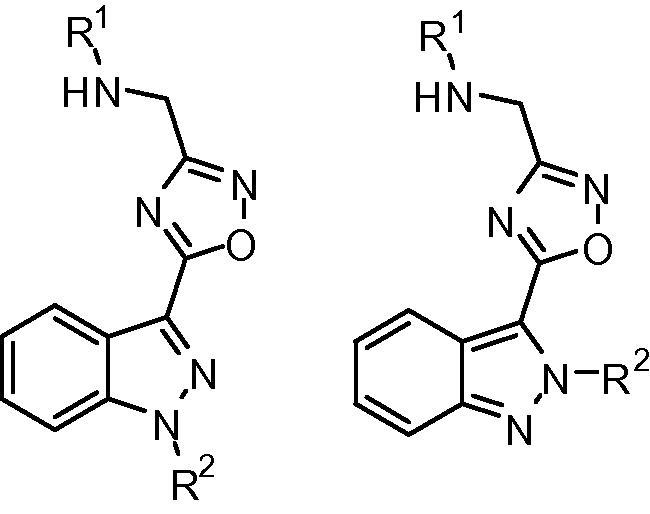

Compound	R^1^	R^2^	cGMP production (pmol/ng protein)	Percent occupancy of sGCcat
**2**	—	—	22.70 ± 3.15	85
**4**	H		14.35 ± 0.11	90
**5**	H		3.22 ± 0.15	101
**6**	H		2.33 ± 0.15	21
**7**	H		9.09 ± 0.31	34
**8**	H		4.05 ± 0.33	20
**9**			2.23 ± 0.06	62
**10**			15.56 ± 1.3	40
**11**	H		2.08 ± 0.01	94
**12**	H		9.11 ± 0.07	96
**13**	H		2.72 ± 0.22	28
DMSO control			2.21 ± 0.15	

**Table 2:** Binding of ATP and YC-1 (**2**) in the presence of GTP

Compound	Biosensor response (RU)	Additive response
ATP (1 mM)	23.04 ± 0.46	
GTP (1 mM)	10.67 ± 0.61	
YC-1 (**2**) (100 μM)	7.10 ± 0.66	
GTP (2 mM) + ATP (2 mM)	25.95 ± 1.00	33.71
GTP (2 mM) + YC-1(**2**) (100 μM)	18.42 ± 1.04	17.77

## Supplementary data

Supplementary data Fig. 1. Binding sensorgrams of YC-1 (A), and Bay 58-2667 (B) to sGCcat (A1, B1,) and full length sGC (A2, B2). At time = 0 s the compound is injected on the protein surface and rapidly binds the immobilised protein, interaction is allowed to take place for 30 s, after which the injection stops and the compounds dissociate from the surface. Fig. 2. Correlation between binding of compounds to the full length and catalytic domain of sGC (A) and between binding and *C* log *P* (B). The sensorgram binding responses were normalised by dividing the response (RU) by the molecular weight of the compound and multiplying by 100.
