# Supplementary material for: Surface plasmon resonance using the catalytic domain of soluble guanylate cyclase allows the detection of enzyme activators
Source: Bioorg Med Chem Lett. 2014 Feb 15;24(4):1075–9. doi: 10.1016/j.bmcl.2014.01.015 (PMC3978654; doi:10.1016/j.bmcl.2014.01.015)
Supplement: Supplementary data — Fig. 1. Binding sensorgrams of YC-1 (A), and Bay 58-2667 (B) to sGCcat (A1, B1,) and full length sGC (A2, B2). At time = 0 s the compound is injected on the protein surface and rapidly binds the immobilised protein, interaction is allowed to take place for 30 s, after which the injection stops and the compounds dissociate from the surface. Fig. 2. Correlation between binding of compounds to the full length and catalytic domain of sGC (A) and between binding and C log P (B). The sensorgram binding responses were normalised by dividing the response (RU) by the molecular weight of the compound and multiplying by 100. [file mmc1.docx]

**Supplementary Data**

# Surface plasmon resonance using the catalytic domain of soluble guanylate cyclase allows the detection of enzyme activators

Filipa Mota^a^, Charles K. Allerston^b^, Kathryn Hampden-Smith^a^, John Garthwaite^a^, David L. Selwood^a^

^a^ The Wolfson institute for biomedical research, University College London, United Kingdom

^b^ Structural Genomics Consortium, University of Oxford, United Kingdom

## Supplementary Figure 1


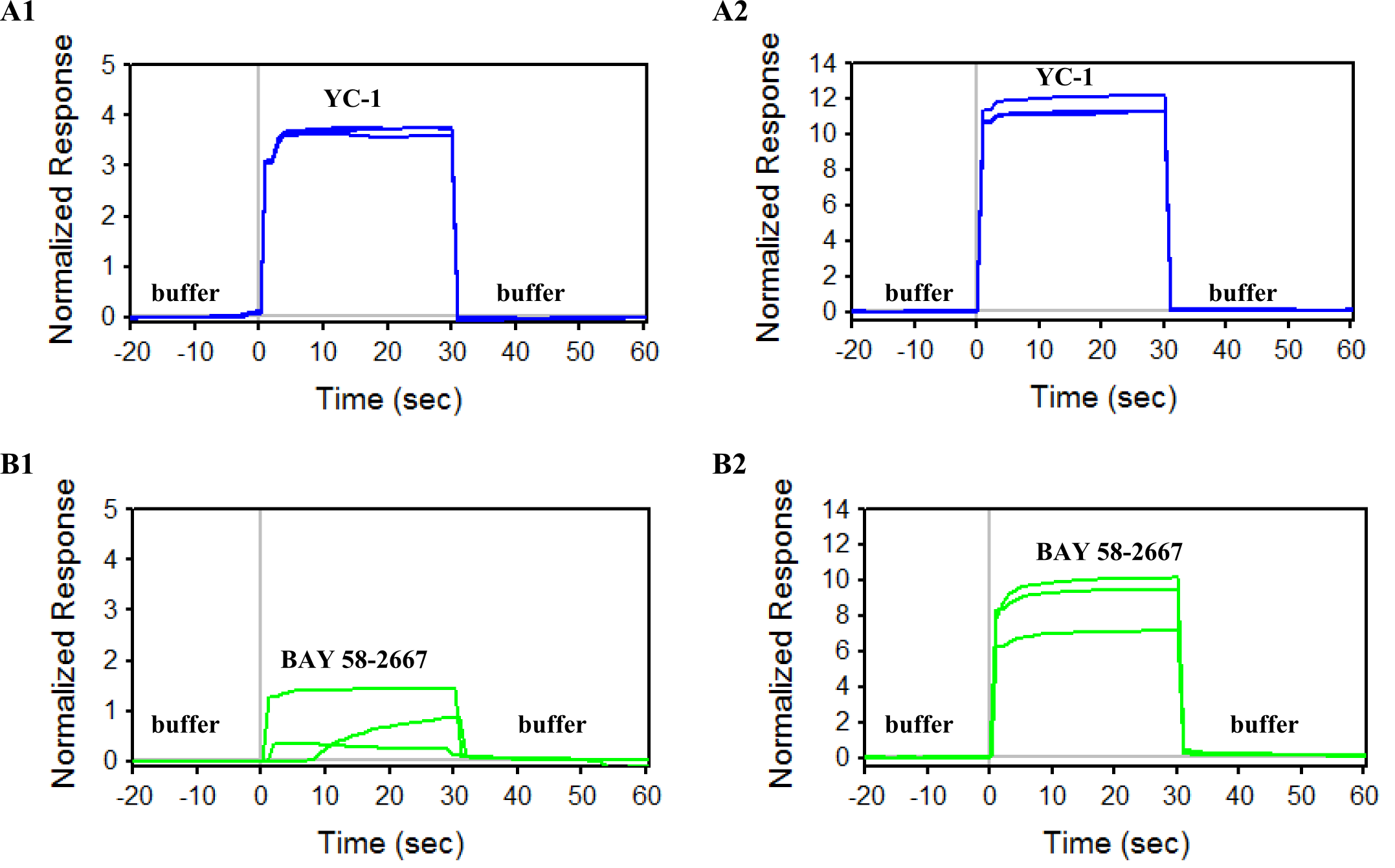


**Supplementary Figure 1.** Binding sensorgrams of YC-1 (**A**), and Bay 58-2667 (**B**) to sGCcat (**A1**, **B1**,) and full length sGC (**A2**, **B2**). At time = 0 sec the compound is injected on the protein surface and rapidly binds the immobilised protein, interaction is allowed to take place for 30 sec, after which the injection stops and the compounds dissociate from the surface.

##

## Supplementary Figure 2

**
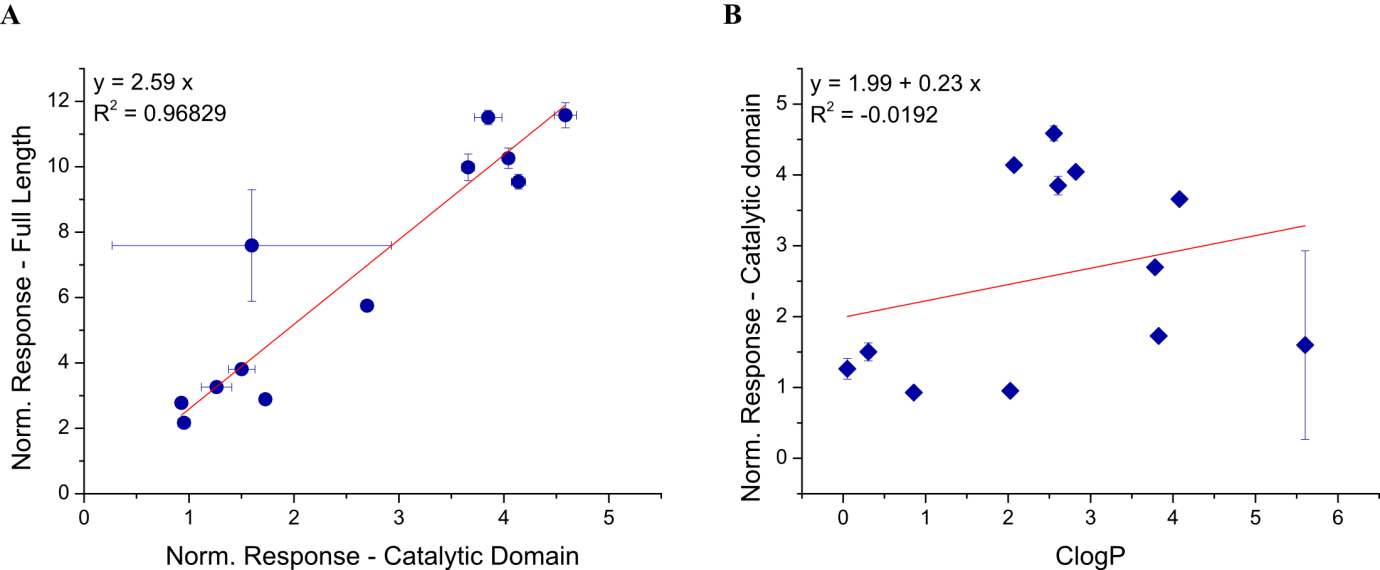
**

**Supplementary Figure 2**. Correlation between binding of compounds to the full length and catalytic domain of sGC (**A**) and between binding and ClogP (**B**). The sensorgram binding responses were normalised by dividing the response (RU) by the molecular weight of the compound and multiplying by 100.
